# Signaling pathways involved in chronic myeloid leukemia pathogenesis: The importance of targeting Musashi2-Numb signaling to eradicate leukemia stem cells

**DOI:** 10.22038/ijbms.2019.31879.7666

**Published:** 2019-06

**Authors:** Foruzan Moradi, Sadegh Babashah, Majid Sadeghizadeh, Arsalan Jalili, Abbas Hajifathali, Hajifathali Roshandel

**Affiliations:** 1 Department of Molecular Genetics, Faculty of Biological Sciences, Tarbiat Modares University, Tehran, Iran; 2 Hematopoietic Stem Cell Research Center, Shahid Beheshti University of Medical Sciences, Tehran, Iran; 3 Department of Stem Cells and Developmental Biology at Cell Science Research Center, Royan Institute for Stem Cell Biology and Technology, ACECR, Tehran, Iran; 4 Taleghani Hospital, Shahid Beheshti University of Medical Sciences, Tehran, Iran; 5 Department of Hematology, School of Medical Sciences, Tarbiat Modares University, Tehran, Iran

**Keywords:** BCR-ABL1, Chronic myeloid leukemia, Cancer stem cells, Signaling pathways, Self-renewal, Targeted therapy

## Abstract

**Objective(s):**

Chronic myeloid leukemia (CML) is a myeloid clonal proliferation disease defining by the presence of the Philadelphia chromosome that shows the movement of BCR-ABL1. In this study, the critical role of the Musashi2-Numb axis in determining cell fate and relationship of the axis to important signaling pathways such as Hedgehog and Notch that are essential for self-renewal pathways in CML stem cells will be reviewed meticulously.

**Materials and Methods:**

In this review, a PubMed search using the keywords of Leukemia, signaling pathways, Musashi2-Numb was performed, and then we summarized different research works***.***

**Results:**

Although tyrosine kinase inhibitors such as Imatinib significantly kill and remove the cell with BCR-ABL1 translocation, they are unable to target BCR-ABL1 leukemia stem cells. The main problem is stem cells resistance to Imatinib therapy. Therefore, the identification and control of downstream molecules/ signaling route of the BCR-ABL1 that are involved in the survival and self-renewal of leukemia stem cells can be an effective treatment strategy to eliminate leukemia stem cells, which supposed to be cured by Musashi2-Numb signaling pathway.

**Conclusion:**

The control of molecules /pathways downstream of the BCR-ABL1 and targeting Musashi2-Numb can be an effective therapeutic strategy for treatment of chronic leukemia stem cells. While Musashi2 is a poor prognostic marker in leukemia, in treatment and strategy, it has significant diagnostic value.

## Introduction

Cancer refers to a group of diseases, which irregular cell growth is their outstanding feature; it can predominantly invade and spread cells from the primary site to other parts of the body (1, 2). When immature blood cells in the bone marrow (progenitor cell or precursor) grow uncontrollably and prevent the production of healthy blood cells, a type of cancer called leukemia is created, which is the unregulated proliferation of cells and disrupting of the bone marrow functions leading to an untimely death if left untreated (3, 4). Leukemia can be acute or chronic; each of them is divided into lymphoid and myeloid lineages according to their origins (5, 6). Chronic myeloid leukemia (CML) is a monoclonal proliferating malignant associated with myeloid lineage (7). CML is diagnosed by detecting increased clonal hematopoietic stem cell(s) (HSCs) that has a shift between chromosomes 9 and 22. The resulting chromosome is called Philadelphia (Ph). The inverted Abelson murine leukemia viral oncogene homolog 1 (ABL1) gene located in 9p34 is placed in breakpoint cluster region protein (BCR) gene located in 22q11 zone and results in the formation of BCR-ABL1 fusion gene that creates tyrosine kinase BCR-ABL1, which is always active and is the activator of several molecular pathways. Ultimately, this gene leads to abnormal cell adhesion, increased proliferation, and inhibition of apoptosis (8-10). CML clinical courses are divided into chronic phase, accelerated phase and blastic phase (8, 11). It develops slowly, while gradually indicates an increase in blood and bone marrow blast percentage, and the accelerated phase will evolve as a blast crisis phase (12, 13), which progressive resistance to therapy happens during this stage (14, 15). 

Tyrosine kinase that is an important mediator of the signaling cascade plays a key role in diverse biological processes like growth, differentiation, metabolism and apoptosis. For decades, it had been postulated that Imatinib mesylate, an inhibitor of the specific BCR-ABL1 tyrosine kinase, is the most effective and selective therapeutic strategy, which had been developed as the first molecularly targeted therapy (16-19). Imatinib typically targets abnormal cells in leukemia, but they are not effective on leukemia stem cells (LSCs) (20). So, these cells remain intact during the conventional cancer therapies, such as chemotherapy and radiation therapy, leading to tumor recurrence and metastasis. Therefore, recognizing the pathways of proliferation, self-renewal, and survival of normal and malignant stem cells can lead to a better understanding of cancer and to find new therapies, especially by targeting cancer stem cells (21).

Accumulating evidence has demonstrated that all of the important signaling pathways related to survival of LSCs can be activated by oncogene BCR-ABL1; therefore, this oncogene creates self-renewal property in LSCs (22, 23). However, BCR-ABL1 oncogene alone cannot be responsible for the self-renewal ability of the committed progenitors for transforming them and will use self-renewal properties of the cells such as HSCs (22, 24, 25). The signaling pathways like Hedgehog (Hh) (26, 27), Wnt/β-catenin (28), Notch (25), Alox5 (30, 31), Foxo (65-67), and Ras (62) may play roles in differentiation and survival of LSCs (35, 36). Beside them, Musashi2 (Msi2)-Numb signaling axis is a molecular pathway, which is correlated with other signaling pathways such as Hh and Notch involved in regulating the self-renewal properties of LSCs (37-39). The Msi2 elimination can increase Numb expression levels. This increment of Numb through the key genes of Hh and Notch signaling pathways can decrease the number of LSCs. Hence, the importance of targeting the Msi2-Numb signaling axis to eradicate LSCs will be discussed in details. Regulating these signaling pathways in cancer cells is disrupted; therefore, study in this area may offer some options in the treatment of leukemia.

## Materials and Methods

The following data was collected by electronic databases, including PubMed, Scopus, ScienceDirect and Cochrane library, Google Scholar, and Web of Science. All types of relevant studies, original articles, books and abstracts were included and their results were reviewed and reported until 2018.

## Results

***The resistance of CML stem cells to BCR-ABL1 kinase inhibitor called Imatinib***

**Figure 1:**
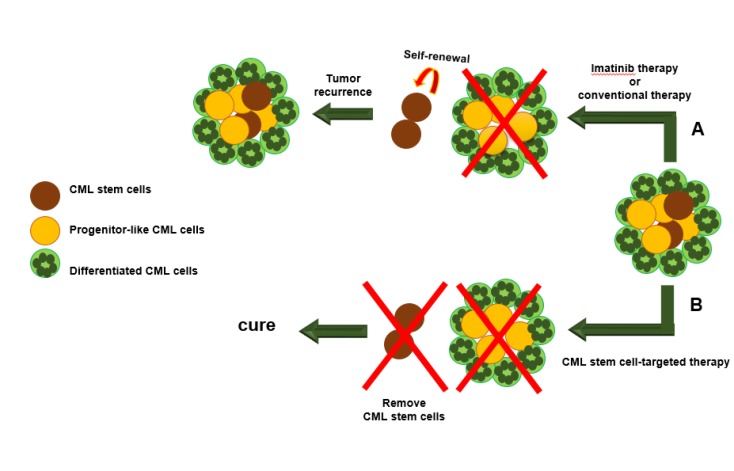
Chronic myeloid leukemia (CML) stem cells are resistant to conventional treatments or breakpoint cluster region protein- Abelson murine leukemia viral oncogene homolog 1 (BCR-ABL) kinase inhibitor called Imatinib. (A) Conventional treatments such as chemotherapy and radiation therapy that target dividing cells and decrease tumor mass cannot prevent tumor regrowth because these approaches are not capable of killing the cancer stem cells. Although Imatinib can inhibit an integrated protein called BCR-ABL1 and decrease the number of CML cells, this inhibitor cannot target the CML stem cells, and this leads to recurrence of the disease. (B) If there would be a method that specifically targets CML stem cells and leads to decrease the self-renewal of CML stem cells, the remaining cells would not be able to support their cancer identity. This approach may avoid drug resistance and recurrence of disease in the patients with CML who are treated with Imatinib. This figure was adapted from reference

The development of Imatinib mesylate, as a tyrosine kinase inhibitor, that is used as a first-line treatment in newly diagnosed CML patients had brought significant successes in the treatment of patients with CML (40-42). Using Imatinib 400 mg/day for patients with chronic disease, led to 70% cytogenetic improvement and 3-year survival. This level is lower in patients with accelerated phase or blast crisis (43, 44). Although continuous Imatinib therapy, especially in CML stage, leads to permanent responses, about 20 to 30 percent of CML patients show resistance to Imatinib therapy and only a few percent will improve and that is because of the secondary mutations in BCR-ABL1 and generating kinases. Patients are resistant to the Imatinib therapy and only a few percent will improve (45). Hence, there is no evidence to show that patients receiving Imatinib can safely stop using it since most of the patients who had interrupted their treatments, have faced with early recurrences of the molecular and cytogenetic level even after remission (43, 44, 46) and therefore these patients have to endure a lifelong therapy (47-50). Conventional treatments such as chemotherapy and radiation therapy that target dividing cells and decrease tumor mass cannot prevent tumor regrowth because these approaches are not capable of killing the cancer stem cells (21). Another problem in conventional treatment procedures is the low specifications, which makes the drugs kill the natural cells beside the tumor cells, and this is one reason for the ineffectiveness and side effects of these methods (51, 52). This is supported since BCR-ABL-positive LSCs remain intact even after long-term imatinib therapy and can cause relapse of the disease. These findings suggest that inhibition of BCR-ABL tyrosine kinase activity alone is insufficient to eradicate LSCs (53-58). Therefore, development of efficient therapeutic strategies capable of targeting key genes involved in self-renewal signaling pathways in LSCs would provide noticeably improved therapeutic benefits to patients suffering CML (59-63) (Figure 1). It was clear that self-renewal property of stem cells can create drug-resistant and recurrence of disease in different cancers (64, 65). It is believed that genetic changes cause deregulation of stem cells that in turn will provide unlimited self-renewal potential for them. As a result, aberrant self-renewal of stem cells provides a prerequisite for initiation, progression, and resistance to cancer (66, 67). Since HSCs and CML stem cells are capable of self-renewal, it is not inconceivable that several signaling pathways that are involved in the regulation of standard stem cells have also roles in cancer stem cell biology, and in this context, there is evidence showing that most of the pathways that are classically associated with cancer may participate in the regulation of normal stem cell development (36, 58, 68, 69). 

**Figure 2:**
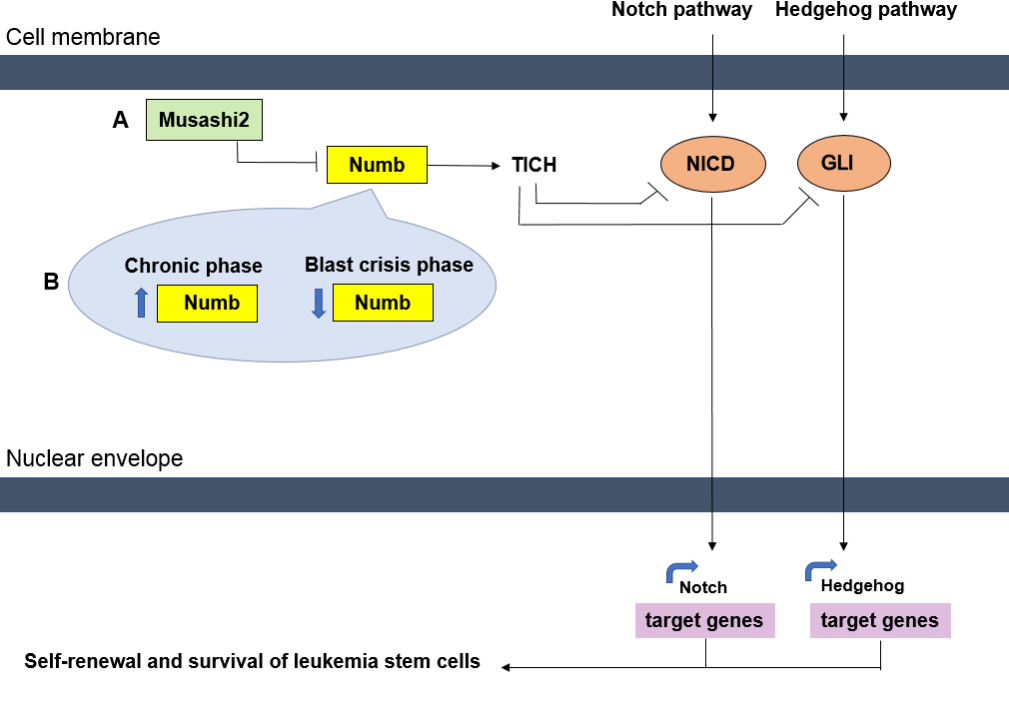
The Musashi2 (Msi2) gene is a major factor in normal hematopoietic and leukemia stem cells. (A) The elevated level of Msi2 leads to the downregulation of the cell-fate determinant, Numb gene, which probably relates to other signaling pathways like Hedgehog and Notch. Numb may decrease the number of Leukemia stem cells via ITCH factor. ITCH ubiquitinates Notch intracellular domain (NICD) and GLi that are respectively the component of Notch and Hedgehog pathways. It is worth noting that Hedgehog and Notch pathways are important pathways that play significant roles in the self-renewal of resistant cancer stem cells. (B) The continued suppression of Numb gene is required to maintain the blast crisis in CML. While the increasing of Numb expression or the inhibition of Msi2 expression might lead to inhibition of the Notch signaling pathway. Therefore, the CML blastic phase is suppressed. This figure was drawn by the writer to summarize the pathways involved in cell fate

**Table 1:** Msi2 expression in human leukemic cell lines. According to the results of Zhang and coworkers [73] Western blotting analyses showed high expression of Msi2 protein in KG-1a and K562 cells, and low expression in U937 and OCI-AML3 cells

**Leukemia type**	**Cell line**	**Msi2 relative expression**
CML	K562	Upregulated
AML	KG-1a	Upregulated
AML	HL-60	Upregulated
AML	THP-1	Upregulated
AML	OCI-AML3	Downregulated
AML	U937	Downregulated

**Table 2:** Msi2 expression in human leukemic cell lines. According to the results of Kharas and colleagues [70] Western blotting analyses showed the expression levels of Msi2 protein in 7 different leukemic cell lines

**Leukemia type**	**Cell line**	**Msi2 relative expression**
AML	Nomo-1	Upregulated
AML	Skm-1	Upregulated
AML	U937	Upregulated
AML	NB4	Upregulated
AML	Mono Mac 6	Upregulated
AML	THP1	Upregulated
AML	OCI-AML3	Downregulated

***Musashi2-Numb signaling axis***

Musashi family involves a group of RNA-binding proteins that are expressed in stem cells and invasive tumors (70). In mammalian, two isoforms of this family called Msi1, and Msi2 have been identified (37). Msi1 gene is located on chromosome locus 12q24 and has 22 exons, and the Msi2 gene has 19 exons and is placed on chromosome locus 17q22. Both isoforms are identified by RNA motifs (RRM), which have high homology in their sequences (72). Experimental investigations show special specification in the binding region of both proteins (71). The similarity in the amino acid surface at the binding region to RNA is 81% for the first RRM and 93% for the second RRM, while 100% similarity is reported for the region of binding, which includes octapeptide conserved sequences (RGFGFVTF) (72). Musashi proteins affect asymmetric cell division, stem cell functions and identifying cell fate in different somatic cells (73, 74). However, their expression patterns in diverse tissues are different. Msi1 in neural stem cells and periventricular progenitor cells in the embryo and also after birth are rich. It is also active in the mammalian brain, gastrointestinal tract, mammary gland, skin, and tumorigenesis (75-82). Overexpression of Msi1 in the advanced stages of the disease and also the prognosis associated with colon and breast cancers (83, 84), urothelial, esophageal, cervical cancer as well as central nervous system tumors, including glioma, medulloblastoma, and ependymoma is recorded (85-90). Some tissues like central nervous system, and nervous stem cells (NSCs) show the simultaneous activity of Msi1 and Msi2 (76). What is important is the point that Msi1 expression in HSCs and progenitor cells are very small and negligible when compared to Msi2 (38, 70), while Msi2 in hematopoietic systems has striking expression (73), and it is recognized as a primary regulator of the HSCs (70, 91) and LSCs (38, 70). Several studies have also reported the relationship of the Msi2 family members to solid tumor pathogenesis (85, 92, 93).

Msi2 gene has been identified as a significant indicator for myeloid leukemia (94). Not only the overexpression of Msi2 is a weak marker in the progression of human CML (38), but it is also related to the rapid progression and poor prognosis in myeloid leukemia (38, 70, 95). Previous studies have shown that Msi2-Numb developmental pathway plays a critical role in the retention of CML stem cell functions and the activities of such pathways would lead to proliferation, development, and survival of LSCs. In other word, Msi2 gene plays a significant role in complex regulation pathways that are involved in self-renewal, and proliferation of HSCs such as Ras, mitogen-activated protein kinase (MAPK), Cycline D1, Myc (70), homeobox genes Meis1, HOXA9, HoX10, Hh and Notch pathways and possibly the other pathways as well (91). Msi2 can control the translation by specific sequence interactions with 3’UTR of target mRNAs in the different stem cells (96). In this regard, studies of Ito *et al.* and Kharas *et al.* in 2010 were the first studies that have shown the relationship between the Msi2 inducer of the Msi2-Numb pathway and hematopoietic malignancy, and their investigations indicated the Msi2 portion and its regulative interactions with leukemia in a stable and definitive manner (38, 70). The findings of these two reports are a primary step in treating aggressive leukemia. Using *in vivo* CML models in chronic phase and blast crisis (13) suggested that Msi2-Numb can be a novel target for leukemia treatment since it can control CML stem cells differentiation and apoptosis (38). In another study, Zhang *et al.* demonstrated that Msi2 knockdown inhibited leukemic cell proliferation and promoted cell apoptosis involving the MAPK signaling pathway. Their study provided novel insight into the mechanisms of leukemogenesis (73). They investigated Msi2 expression at protein levels in K562, KG-1a, HL-60, THP-1, OCI-AML3 and U937 cell lines. According to their study, western blotting analyses showed high expression of Msi2 protein in KG-1a and K562 cells, and low expression in U937 and OCI-AML3 cells (Table 1). Kharas et al. showed high Msi2 expression levels in 6 cell lines associated with acute myeloid leukemia (AML) called Nomo-1, Skm-1, U937, NB4, Mono Mac 6, THP1 and low Msi2 expression levels in OCI-AML3 cell line (Table 2). The data are provided in the Tables according to references (70, 73). 

Kharas *et al.* analyzed the gene expression of 436 patients with AML and reported that Msi2 expression level (as an independent prognosis marker) is related directly to decreased survival (70). They also reported deregulation of the key genes that control the self-renewal and cell fate in HSCs and may play a critical role in leukemia progression, and one of these adverse regulations is associated with Msi2-Numb signaling axis (70). This axis is significantly involved in regulating the cell self-renewal properties (37-39). They tested Msi2 expression inducer called doxycycline both in *in vitro* and *in vivo*; they observed that Msi2 expression in laboratory is at the highest level during the time of forming the hematopoietic colonies with immature myeloid phenotype, and Msi2 expression *in vivo* caused expansion of HSCs and short-term progenitor cells. They also conjugated Msi2 inducer doxycycline with BCR-ABL1 oncogenes and injected to mice. Consistent with other reports, they observed that Msi2 in immature myeloid leukemia in which blast crisis had been reconstructed was induced. Following these studies, expression of this gene in human samples with CML was also investigated (33 patients in blast crisis phase and 57 cases in chronic phase) and the results showed that Msi2 expression at progressive CML stages (blast crisis) is placed on the higher levels to primary levels of CML (chronic phase) and this overexpression of Msi2 has an inverse relationship with the Numb gene in the both blast crisis and chronic phase. Ito and coworkers (38) also indicated that there is a relationship between overexpression of Numb and decreasing of the leukemia cells in mouse models; they suggested that Numb cannot disperse the disease considerably. These results indicated that Numb levels might prevent the progression of CML and induce differentiation in leukemia stem cells. Besides inducing the Numb expression, the results showed that Msi2 gene inhibition by shRNA1 would significantly decrease *in vivo* leukemia growth and retention rate, especially in blast phase. Similar to Numb inducing, Msi2 inhibition can also induce differentiation in leukemia cells and inhibit the ability of proliferation and distribution. A possible mechanism that is important in this area is that the inhibition effects of Msi2 elimination of LSCs may be related to Numb regulative effects, which are a determinant factor in cell fate. The Msi2 elimination can increase Numb expression levels and Numb may remove CML stem cells. These findings are possibly related to other signaling pathways that finally will decrease the numbers of LSCs. 

Msi2 can inhibit the translation by binding to 3’UTR of Numb mRNA. By inhibiting the Msi2, the Numb gene will increase, and this increment of Numb is related to other signaling pathways like Hh and Notch. Finally, it will decrease the number of LSCs. By ubiquitination and destruction with the proteasome, Numb can inhibit Notch intracellular domain (NICD) and Gli that are respectively the components of Notch and Hh pathways. It is worth noting that Hh and Notch pathways are two important pathways that play significant roles in the self-renewal of resistant cancer stem cells. Therefore, by inhibiting these molecular ways, it is expected that cancer cell growth will be decreased and LSCs can be directed to apoptosis (38, 70, 74, 95) (Figure 2 A). Ito *et al*. in the mouse models that were in blastic phase indicated that Numb dramatically expressed at lower levels, while Msi2 expression in these models was particularly high (70). Kharas *et al.* presented the Numb gene expression at the protein levels (with Immunoblot) in LAMA-84 cell line (which is a cell line derived from blastic phase of CML patients) after transfection of siRNA against Msi2 (70). In their study, the inverse relationship between Numb and Msi2 genes was clearly evident. These reports indicate an inverse association of Msi2 gene and gene expression levels of Numb.

***Hedgehog signaling pathway***

Hh signaling pathway was first identified in Drosophila for patterning the early embryo. Several studies show that Hh signaling pathways may regulate the cell fate and maintain the stem cell/progenitor cells (97, 98). This highly conserved developmental pathway is responsible for regulating the normal HSCs and CML stem cells (98-101). Hh pathway is active in the embryonic hematopoiesis, but its level will decrease after birth; nevertheless, this channel will be increased again in patients with CML. This pathway is recognized as a functional pathway in the LSCs and any disruption in this way would prevent CML progression. The importance of Hh signaling pathway in carcinogenesis is attributed to self-renewal of stem cells. Hh pathway is activated by binding the protein ligands (Sonic Hedgehog (Shh), Desert Hedgehog (Dhh) and Indian Hedgehog (Ihh)) (that all of them are secreted glycoproteins (7)) to membrane receptor called Patched (PTCH). PTCH is a negative regulator of another membrane receptor called smoothened (Smo). During the ligand binding, PTCH inhibition effect on Smo is removed. This event causes alteration of Smo conformation and consequently induction of Gli transcription factors (Gli1, Gli2, and Gli3) will occur. The activity of these transcription factors promotes the transcription of Hh genes, such as Gli1, PTCH, CyclinD1 and Bcl-2 (26, 100-102). The role of Hh signaling pathway (particularly Sonic Hh) in regulating the self-renewal was identified when it was discovered that human HSCs indicate increased self-renewal in response to Sonic Hh stimulation in the laboratory, of course with other growth factors (36, 103). Study of Zhao and coworkers (104) showed that Smo elimination might disturb the ability of HSCs self-renewal and decrease the CML induction by BCR-ABL1 oncoprotein. The Smo elimination was also evacuated in the CML stem cell population, while overexpression of Smo can increase the population of stem cells and facilitate the progression of the disease. The possible mechanism is that inhibitory effects of Smo elimination of LSCs may be related to Numb regulatory effects, which evacuate the CML stem cells. 

***Notch signaling pathway***

Notch signaling pathway plays a role in regulating most of the cellular processes like development and regeneration of mature tissues. Notch membrane receptors are also part of the signaling pathways that are essential for regulating the cell fate of the different tissues; for this reason it is suggested that a disruption in Notch signaling pathway may lead to a dysregulation in the genes that are involved in the self-renewal of the stem cells and finally would cause carcinogenesis and oncogenesis (105). In mammals, Msi function for activating the Notch signaling pathway by suppressing the Numb is reported (106). For the first time, Ito *et al.* pointed that in the CML mouse models, Msi2/Numb expression level and Notch signaling pathway may alter and there is a relationship between them. They also mentioned that the continued suppression of Numb gene is required to maintain the blast crisis in CML. While, the increasing of Numb expression or the inhibition of Msi2 expression might lead to inhibition of the Notch signaling pathway and direct the cell toward differentiation; therefore, the CML blastic phase is suppressed. The new findings of the Msi2/Numb/Notch signaling pathway may be a key to understanding a new insight to hematopoietic malignancies, especially in the advanced stages of CML blastic crisis (38, 95) (Figure.2 B). Other studies explained the relationship between Notch signaling pathway and the gene for the route (Hes1) with CML displayed progress (107). According to these results, each family member of Musashi is a key regulator of many intracellular signaling pathways and can control the cell fate and stem cell self-renewal, differentiation, and tumorigenesis. Further studies on the relationship between Msi2 and signaling pathways in cancer and various malignancies, including CML is required. Therefore, the expected asymmetric induction of differentiation into mature cells by regulating the signaling pathways involved in self-renewal of cancer cells may provide the new prospect of therapies for leukemia, especially in the advanced stages (37). 

## Discussion

***Inhibition of Msi2 and its ability to reduce the proliferation, cell cycle arrest and induction of apoptosis in leukemia cells***

Msi2 gene is proposed as a determinant of cell fate since it induces the rise of cell cycle progression in normal and malignant hematopoietic cells. Zhang *et al.* through MTT assay and Colony formation assays showed that the inhibition of Msi2 reduces duplication and the number of cell clones in the K562 cell line (CML) (73). They reported that Msi2 inhibition leads to an increase of K562 cells in G0/G1 phase and reduction of cells in S phase. The suppression of cell cycle by increasing P21 and decreasing the expression of Cdk2 and Cyclin D1 in both mRNA and protein levels following by inhibiting Msi2 were also demonstrated by them. An increase of P21 expression levels stops cell cycle in G0/G1 phase and causes inhibition of cellular transport from G0/G1 to S phase. In another study, de Andres-Aguayo *et al.* and Hope *et al.* showed that inhibition of Msi2 by increased expression of P21 can significantly reduce mouse HSCs in phase S-G2/M (91, 108). Following the blocking of Msi2 gene, Zhang *et al.* showed early apoptosis induction by flowcytometry staining with Annexin-PI, and the late apoptosis through staining with Wright-Giemsa (for detection of apoptotic cell morphology) (73). To confirm the results of induction of apoptosis, they also reported the reduction in Bcl2 gene expression (an important anti-apoptotic factor) and increase in Bax gene expression (an important factor of apoptosis) in both mRNA and protein levels in K562 cell line. Another study by Kharas *et al.* also showed that Msi2 inhibition by shRNA reduces cell proliferation and significantly induces apoptosis of Nomo-1 and THP-1 (AML), the LAMA-84 and AR230 (CML) cell lines (70). 

***The importance of targeting the Musashi2-Numb signaling pathway to eradicate leukemia stem cells***

CML is a malignant disease having chronic and blast crisis phases. More recently, appropriate therapies such as kinase inhibitors, Imatinib, were used in clinical base, but this process is not always a successful treatment for patients who are resistant to the medication. As a result, treatment with this drug has some limitations, so new molecular treatment methods are considered (109). Increasing of Msi2 expression levels in advanced stages of CML in comparison with chronic phase of CML patients predicate the increase of unusual activities of the Msi2-Numb signaling pathway; this expanded expression also has been reported in AML. So, inhibition of another purpose apart from BCR-ABL1 (such as Msi2 as an essential gene in the Msi2-Numb) can be considered as a practical approach to eradicate LSCs. This approach by targeting essential genes in signaling pathways that are associated with self-renewal such as Hh and Notch can cause a reduction in aberrant self-renewal of stem cells in CML and prevent the recurrence of the disease (37, 38, 70, 108).

## Conclusion

In recent years, considerable progress has been made for treatment of patients with CML. However, providing an approach that is specifically able to reduce LSCs held promise for the effective treatment of CML. The inappropriate activity of Msi2-Numb pathway in LSCs has been reported. Imatinib in the treatment of patients with CML has a significant role, but in spite of this, it has been observed that there are still BCR-ABL1 positive cells that Imatinib is unable to target them; these cancer stem cells (CSCs) are believed to be the cause of resistance to Imatinib therapy. Msi2 is a factor that is expressed at the high levels in CSCs; Msi2 inhibition leads to an increase in the Numb gene expression pattern (as the gene that determines cell fate). This increment of Numb through the key genes of Hh and Notch signaling pathways can decrease the number of LSCs. Therefore, it is obvious that inhibition of Msi2 may be a promising therapeutic approach in reducing human LSCs in CML patients resistant to the drug.
